# Retained palmar foreign body presenting as a late hand infection: proposed diagnostic algorithm to detect radiolucent objects

**DOI:** 10.1186/1754-9493-7-25

**Published:** 2013-07-11

**Authors:** Kyros Ipaktchi, Andrew DeMars, Jung Park, Christopher Ciarallo, Meryl Livermore, Rodrigo Banegas

**Affiliations:** 1 Department of Orthopaedic Surgery, Denver Health Medical Center, 777 Bannock Street, Denver, CO 80204, USA; 2 Department of Anesthesiology, Denver Health Medical Center, 777 Bannock Street, Denver, CO 80204, USA; 3 University of Colorado School of Medicine, Anschutz Campus, Aurora, CO 80045, USA

**Keywords:** Foreign objects, Midpalmar space, Ultrasound

## Abstract

**Background:**

Penetrating injuries to the hand can compromise important anatomic structures, and persisting foreign objects may become a source of infection. Foreign body intrusions into the hand are among the most common injuries to the upper extremity seen in the Emergency Department. Radiolucent organic objects, as well as a few higher density inorganic materials such as plastic, present a diagnostic challenge and are routinely missed using standard radiography. While the literature describes the use of high-frequency ultrasound as an adjunct to conventional diagnostics, to our knowledge, no formal algorithm has been published.

**Case presentation:**

We describe a case of incomplete wooden splinter removal, presenting as a late midpalmar abscess five months after the initial injury, and requiring two subsequent surgical explorations for definitive treatment. This case has led us to implement a formal diagnostic pathway including high-frequency ultrasound at our institution. We contrast this presentation with a subsequent case involving a much smaller wooden palmar foreign body that was easily identified under ultrasound and removed without sequelae.

**Conclusion:**

Many hand injuries are caused by low density, radiolucent foreign bodies. These objects can easily escape traditional evaluation in the emergency room including standard radiography. We present an algorithm implementing high frequency ultrasound to minimize the risk of missing radiolucent penetrating foreign objects in the hand.

## Background

Hand injuries are among the most frequent causes for Emergency Department visits, many of which are due to foreign body intrusion [1]. The density of important anatomic structures explains the increased rate of complex injuries and long-term morbidity. Standard physical hand examination fails to detect up to 38% of foreign bodies, and routine radiographs identify wooden objects in only 15% of cases [2,3]. Overall detection of foreign bodies of any material by plain film radiography has been quoted at 80% [4]. In contrast, high-frequency ultrasound (≥ 7.5 MHz) has been shown to identify foreign bodies with a sensitivity of 87-93% and a specificity of 89-99% [5]. Failure to detect organic foreign bodies can lead to infection and functional morbidity. Plant thorns are of particular concern since they have low structural density and high radiolucency, and persisting thorns may lead to chronic synovitis [6].

### Case presentation

#### Case # 1

A 39 year-old right hand dominant male, presented to an outside facility after a large wooden splinter had pierced through the hypothenar eminence of his non-dominant hand and had broken off. Per report, a splinter was removed and plain film radiography was unremarkable. However, despite the procedure, the patient reported a persisting density in his palm. Eight weeks later, the patient presented to our Emergency Department complaining of a “popping” sensation, palmar erythema and swelling in the affected hand. Clinically, the hand was neurovascular intact, with intact tendon function. Forced passive extension was painful in the palm and there was obvious local swelling. The patient had a mild leukocytosis, while C-reactive protein and sedimentation rates were normal. Plain film radiographs were unremarkable except for a well-healed fifth metacarpal fracture and the presence of palmar soft tissue swelling.

**Figure 1:**
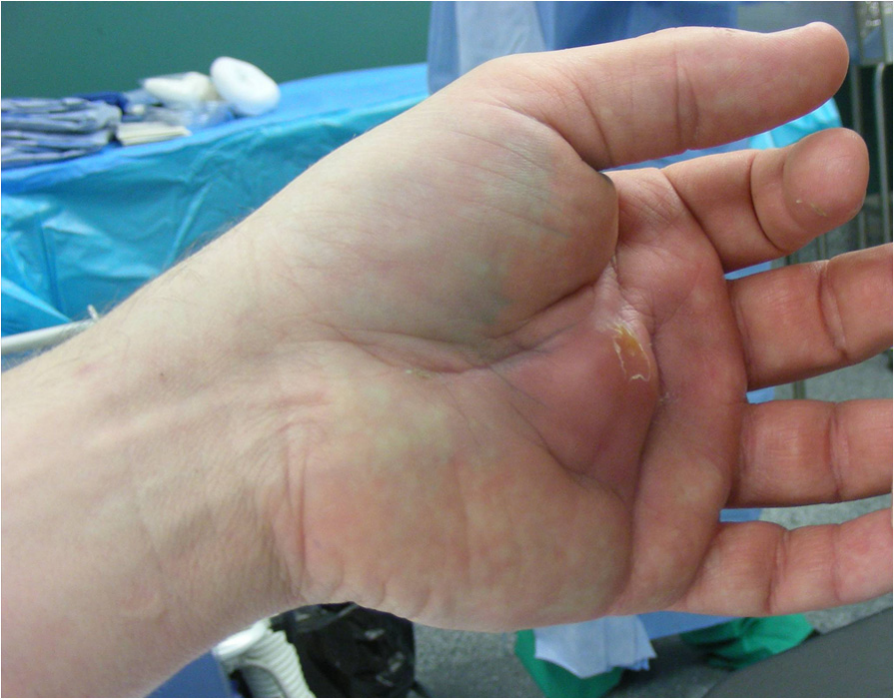
**Intraoperative photo showing a deep palmar abscess.** Five months after incomplete wooden splinter removal the patient is readmitted for surgical exploration. Fingers are held in protective flexion position due to compartmental swelling of the deep palmar space.

**Figure 2:**
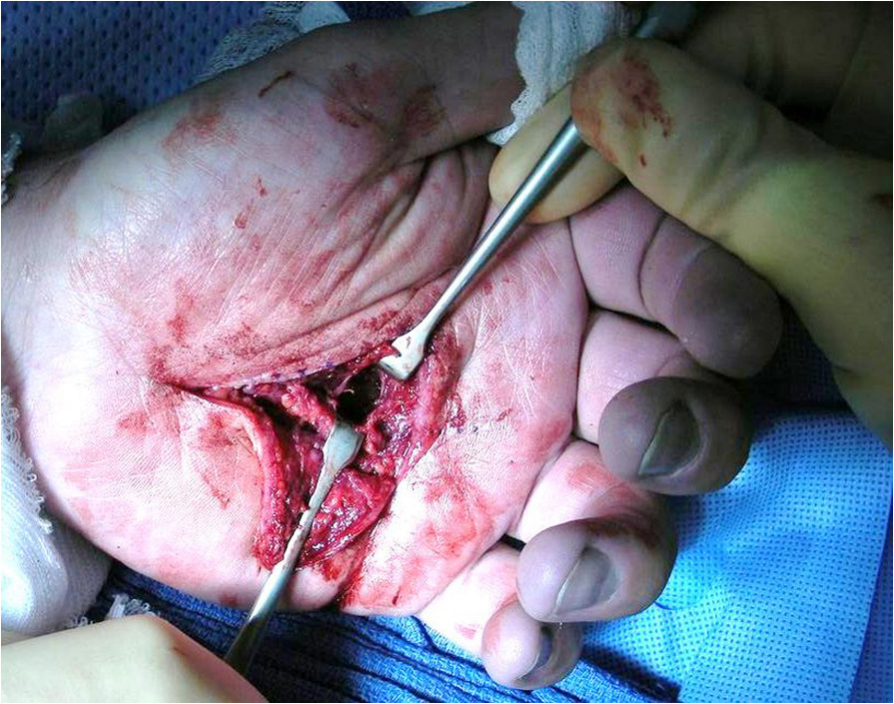
**Opened deep palmar space.** Surgical exploration of the mid-palmar space abscess which had decompressed through the flexor tendons and palmar aponeurosis.

**Figure 3:**
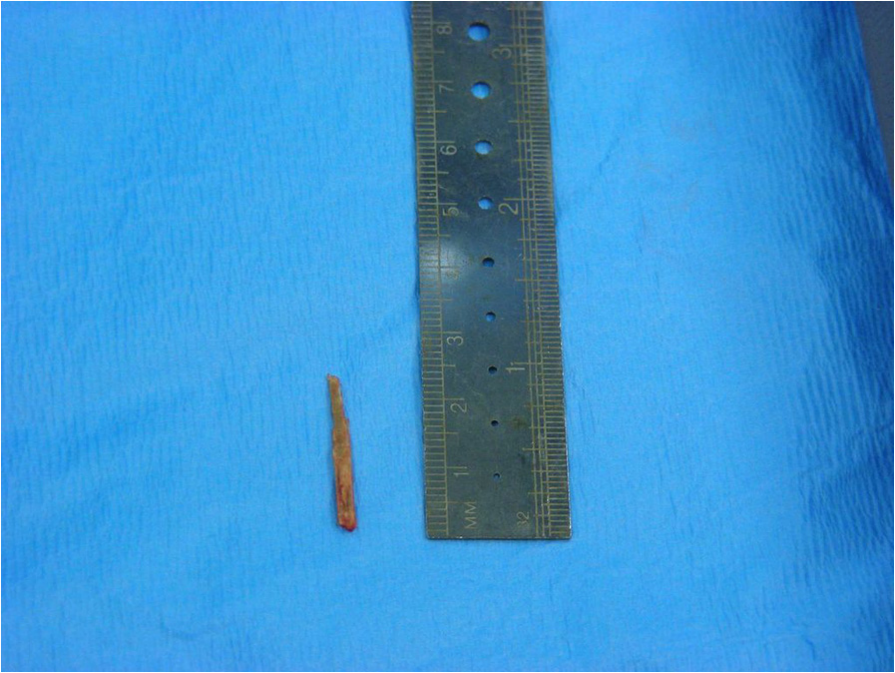
**Retrieved radiolucent one**-**inch long wooden splinter.** The operation yielded the surprising finding of a near 1 inch long wooden splinter which had resided for 5 months undetected in the deep palmar space.

**Figure 4:**
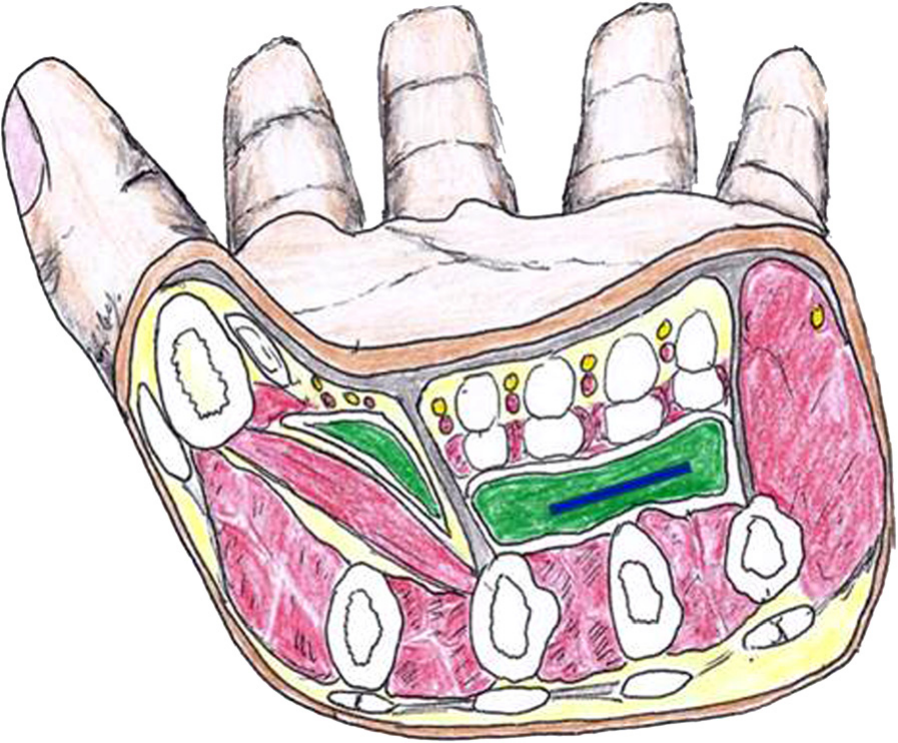
**Drawing of the midpalmar crossectional anatomy.** Green colored thenar and mid-palmar spaces demonstrating the relative depth of these enclosed compartments which can be sites of infection. The location of the removed wooden splinter is depicted in blue.

The patient was taken to the operating room for irrigation and debridement. Exploration of his palm revealed no foreign body or purulence. However, there was inflammation involving the flexor tendon sheath. After an uneventful recovery, the patient regained full finger function and resumed work. Three months later, the patient returned to the Emergency Department with a fluctuant abscess in the affected palm (Figure 1). Surgical exploration at that time showed a large epifascial abscess, which originated in the mid-palmar space and had spread superficially (Figure 2). Further deep exploration revealed, surprisingly, a retained one-inch long wooden splinter (Figure 3). The deep location of the splinter below the flexor tendons in the midpalmar space may have contributed to the failed detection of this object during the first exploration (Figure 4). Subsequent recovery after removal of the foreign body was uneventful.

#### Case # 2

**Figure 5:**
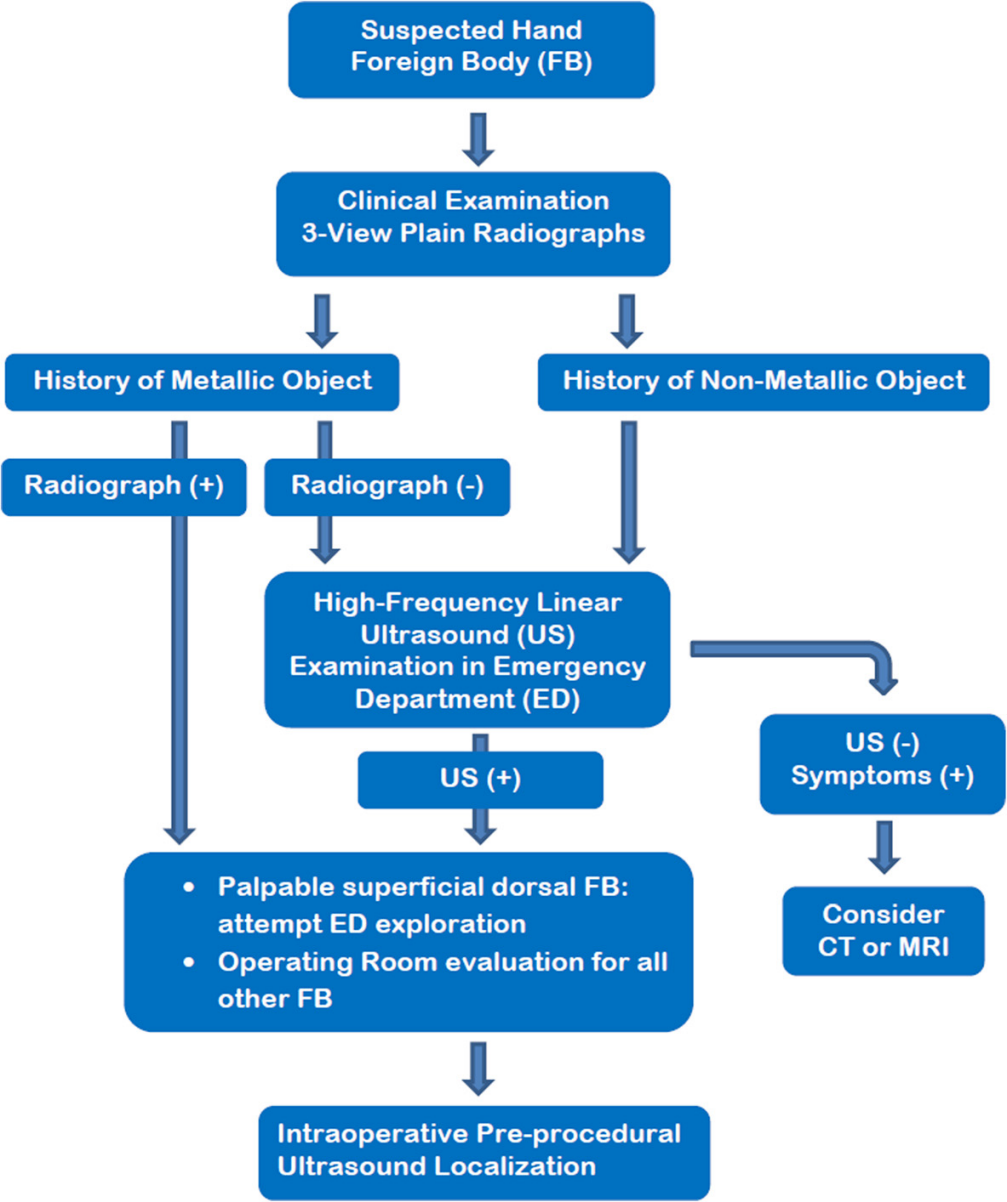
**Proposed diagnostic algorithm to enhance the detection of radiolucent foreign objects in the hand.** This algorithm details the use of high frequency ultrasound in the Emergency room as well as in the operative suite.

**Figure 6:**
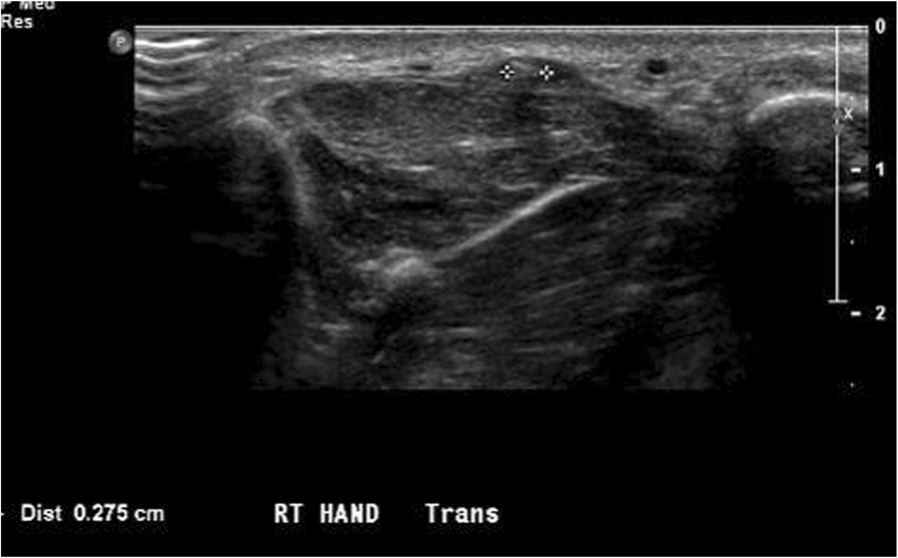
**High frequency linear ultrasound image.** Ultrasound detection of a 2.7 mm wooden splinter in the first webspace distance measurement in between the 2 tracking cross marks. This demonstrates the resolution of current ultrasound probes which would have allowed for easy detection of the nearly 3 cm or 1 inch long splinter shown in Figure 3.

A 46 year-old carpenter had sustained a wooden splinter injury six months before presenting in hand clinic with tenderness in the first webspace. There were no signs of infection on clinical exam and plain film radiographs were negative. Our new foreign body diagnostic pathway was implemented and the high-frequency ultrasound examination revealed a 2.7 mm foreign object in the first webspace (Figures 5 and 6). Subsequent surgical exploration revealed a retained wooden splinter, and the patient had an uneventful recovery.

## Discussion

Common low-density organic materials - such as thorns, wood and fish bones - along with some higher density inorganic materials - such as glass and plastic - account for a large percentage of hand foreign bodies. Historically, soft tissue radiography has been described as a useful diagnostic adjunct for foreign body localization [7]. However, as radiodensity is a consequence of both material density and atomic number, wood is consistently elusive on plain film radiography [2].

Soon after its clinical introduction, computerized tomography (CT) was found to be a highly useful modality to detect deep and embedded low density objects [8]. Although computerized tomography is readily available in most hospitals, it is typically not performed as a dynamic study, and the exposure to ionizing radiation is significant. Magnetic resonance imaging (MRI) has also been advocated for soft tissue foreign body localization. However, there are significant financial costs and long acquisition times, and extremity MRI protocols are frequently limited to two millimeter section intervals. Additionally, magnetic resonance imaging may produce an unacceptable number of false-positive diagnoses, as structures such as scar tissue, tendons and calcifications may be mistaken for foreign bodies [9].

Ultrasound has been used for foreign body localization since 1978 [10]. While the ultimate resolution limit of a 10 MHz sound wave in tissue is approximately 0.15 mm, practical application of high-frequency ultrasound has identified foreign objects as small as 0.5 mm and as deep as 4 cm [3]. The use of ultrasound conducting gel as a standoff pad or immersing the extremity in a water bath during visualization have both been described to improve the detection of superficial foreign bodies and to minimize patient discomfort from direct transducer pressure [11]. Dynamic ultrasound scanning should be performed, and the tissue should be imaged in two planes in an axis perpendicular to the surface of the suspected foreign body.

Most foreign bodies are hyperechoic, and the induced inflammatory changes appear as a surrounding hypoechoic rim, beginning within twenty-four hours of intrusion [5]. Metal and glass may cause a “comet tail” artifact, while gravel demonstrates strong posterior acoustic shadowing [11]. Power Doppler may be used to identify acute inflammatory changes and neogranulation around foreign bodies, but it may not be positive for up to two days after intrusion [12]. Limitations to ultrasound detection of soft tissue foreign bodies include gas bubbles, hematomas, calcified soft tissues and operator inexperience. While some authors have argued that high-frequency ultrasound should be the imaging modality of choice for foreign body localization, others have suggested that ultrasound should replace conventional fluoroscopy for preoperative localization [3].

## Conclusion

Many hand injuries are caused by low density, radiolucent foreign bodies. Superficial foreign bodies can frequently be localized during physical examination and removed without difficulty. However, radiolucent objects, particularly those that are smaller and deeper, may not be identified on plain film radiographs or even during superficial surgical exploration. As compared to other soft tissue imaging modalities such as CT and MRI, high-frequency ultrasound appears to be an equally efficacious, more cost-effective, and readily available option in most Emergency Departments. Additionally, high-frequency ultrasound allows for a dynamic, real-time intervention with concurrent evaluation of adjacent soft tissues. With the intent to optimize foreign body detection, minimize cost and reduce exposure to ionizing radiation, we present a formal diagnostic algorithm that includes ultrasound to evaluate suspected hand foreign bodies.

### Consent

Written informed consent was obtained from the patient for publication of this Case report and any accompanying images. A copy of the written consent is available for review by the Editor-in-Chief of this journal.

## Abbreviations

MHz: Megahertz; Mm: Millimeter; CT: Computerized tomography; MRI: Magnetic resonance imaging; US: Ultrasound

## Competing interests

The authors declare that they have no competing interests.

## Authors’ contribution

KI provided the case and idea and prepared the manuscript, AD and JP reviewed the manuscript, CC reviewed of manuscript and provided the ultrasound content and designed Figure 6 together with KI; RB reviewed the manuscript and drew Figure 4, ML reviewed the manuscript. All authors read and approved the final manuscript.
